# *Agemone mexicana* flavanones; apposite inverse agonists of the β2-adrenergic receptor in asthma treatment

**DOI:** 10.6026/97320630014060

**Published:** 2018-02-28

**Authors:** Gabriel O. Eniafe, Damilohun S. Metibemu, Olaposi I. Omotuyi, Adewale J. Ogunleye, Olumide K. Inyang, Niyi S. Adelakun, Yakubu O. Adeniran, Bamidele Adewumi, Ojochenemi A. Enejoh, Joseph O. Osunmuyiwa, Sidiqat A. Shodehinde, Oluwatoba E. Oyeneyin

**Affiliations:** 1 Centre for Biocomputing and Drug Development, Adekunle Ajasin University, Akungba Akoko, Ondo State, Nigeria; 2 Department of Biochemistry, Adekunle Ajasin University, Akungba Akoko, Ondo State, Nigeria; 3 Department of Chemical Sciences, Adekunle Ajasin University, Akungba Akoko, Ondo State, Nigeria

**Keywords:** Asthma, β2- adrenergic receptor, Inverse agonists, Flavanone

## Abstract

Asthma is an inflammatory disease of the airway that poses a major threat to human health. With increase industrialization in the developed and developing countries, the incidence of asthma is on the rise. The β2-adrenergic receptor is an important target in designing anti-asthmatic drugs. The synthetic agonists of the β2-adrenergic receptor used over the years proved effective, but with indispensable side effects, thereby limiting their therapeutic use on a long-term scale. Inverse agonists of this receptor, although initially contraindicated, had been reported to have long-term beneficial effects. Phytochemicals from Agemone mexicana were screened against the human β2-adrenergic receptor in the agonist, inverse agonist, covalent agonist, and the antagonist conformations. Molecular docking of the phyto-constituents showed that the plant constituents bind better to the inverse agonist bound conformation of the protein, and revealed two flavanones; eriodictyol and hesperitin, with lower free energy (ΔG) values and higher affinities to the inverse agonist bound receptor than the co-crystallized ligand. Eriodictyol and hesperitin bind with the glide score of -10.684 and - 9.958 kcal/mol respectively, while the standard compound ICI-118551, binds with glide score of -9.503 kcal/mol. Further interaction profiling at the protein orthosteric site and ADME/Tox screening confirmed the drug-like properties of these compounds.

## Background

**Figure 1:**
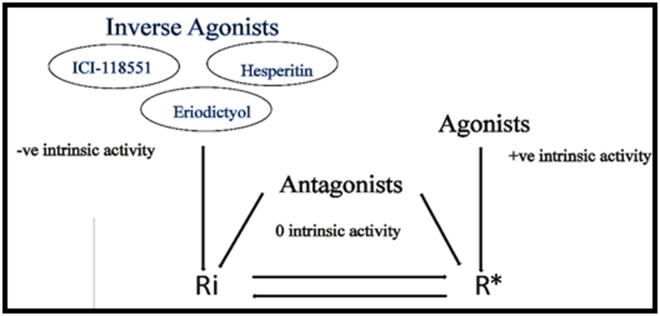
Schematic diagram of the receptor active (R*) and inactive (Ri) states in equilibrium, showing the preferential affinity of agonists to the active state, inverse agonists to the inactive state and antagonists to either of the receptor states, with positive, negative and zero intrinsic activities respectively.

Asthma, a chronic disease of the airway, is one of the leading cause of mortality throughout the world which has increased in severity in the past few decades, both in developed and developing countries. Atopic patients show symptoms of wheezing, acute exacerbation of cough, chest tightness, forced expiratory volume and reduced airflow. The morbidity, mortality and increased prevalence rate of asthma in recent years display the grave upsurge and severity of the disease in the human population. About 300 million people have been diagnosed of the disease, and a further 100 million people are estimated to be affected by 2025 [1]. Hence, an expedient research with a high result-yielding stance is pertinent in the effective management of the disease. The β2-adrenergic receptor (β2-AR) belongs to the Gprotein coupled receptor (GPCR) family, which is coupled to a heterotrimeric stimulatory G-protein (Gs-protein) to activate downstream effectors, such as adenylyl cyclase, phospholipase C etc. β2-AR consists of 7-transmembrane alpha helices which are connected by three extracellular loops and three intracellular loops. The receptor structure consists of an extracellular N terminus and an intracellular C-terminus [2]. Like other GPCRs, β2-AR can undergo conformational changes on ligand binding, and exist in multiple states. According to the two-state receptor model, a receptor remains in equilibrium i.e. an inactive (Ri) and active (R*) state (Figure 1). This equilibrium is altered when a ligand binds at the receptor active site, favouring either R* (activation) or Ri (stabilization). Agonists preferentially bind to R* with positive intrinsic activity or efficacy, while antagonists bind either of the receptor states with zero efficacy. Inverse agonists are uniquely recognised for stabilizing constitutively active receptor. They bind preferentially to Ri with intrinsic activity below zero.

Over the years, synthetic agonists of the human β2-AR seem to be saviours to asthma patients from the intermittent life-threatening horror of the disease, however, various side effects of these drugs have been reported e.g. Salbutamol, the common asthmarelieving drug, causes muscle tremors, palpitation, restlessness, tachycardia etc [3]. Other than these observed side effects are the deleterious consequences of the chronic administration of these agents, resulting in the majority of asthma related death [4]. Consequently, the quest for drug(s) safe in the palliative management of the disease is logical. The acute beneficial and chronic deleterious effect of the β2-AR agonist is mirrored by the acute deleterious and chronic beneficial effect of inverse agonist of the receptor [5]. Although initially contraindicated, β2-AR inverse agonists have been found effective in the chronic treatment of asthma [5].

Agemone mexicana, also known as yellow thistle was listed as one of the plants that exhibit anti-asthmatic potential [6]. Brahmachari et al. [7] reported different classes of compound, such as the alkaloids, terpenoids, steroids, carotenoids and flavonoids present in the plant. Flavonoids are major polyphenolic plant metabolites that are found in various human foods, and have been reported to possess anti-asthmatic property [8]. Flavonoids are further classified as flavones, flavonols, isoflavones, anthocyanidins, flavanols and flavanones [9]. In the present study, two flavanones, Eriodyctyol (ERI) and Hesperitin (HES) were identified as potent apposite agents for the treatment of asthma, exhibiting inverse agonism.

## Methodology

The Schrodinger small molecule drug discovery suite, version 2017-1 [10] and AutoDock Vina [11] were used in this study.

### Protein Preparation

Owing to the different bound conformations of GPCRs, we retrieved the 3D structures of the human β2-AR bound to an agonist, 4LDE [12], covalent agonist, 4QKX [13], inverse agonist, 3NY8 [14], antagonist, 3NYA [14] and another inverse agonist bound (also the inactive state of the receptor), 5JQH [15] conformations from the Protein Data Bank (www.rcsb.org). Protein structures were viewed on the Schrodinger Maestro 11.1, version 2017-1. Lysozyme, water molecules as well as other noninteracting ligands were removed. A complete protein preparation was performed using the Protein Preparation Wizard. The grid coordinates were respectively generated around the corresponding crystallized ligands of the various protein conformations (Table 1).

### Ligand Library and Preparation

Phytochemicals characterized from Agemone mexicana were obtained from literatures and a library of compounds from Agemone mexicana was created. The compounds were downloaded in the structure-data file (sdf) format from the NCBI PubChem database (https://www.ncbi.nlm.nih.gov/pccompound). The ligand library was prepared for docking using AutoDock Vina and Glide. The Babel and MGLTools were used for ligand preparation for AutoDock Vina, while the Schrodinger Ligprep was used for Glide docking.

### Molecular Docking of Ligands

Virtual screening of the ligand library was performed against the five prepared protein structures using AutoDock Vina compiled under Ubuntu 14.04 LTS. However, the flavonoid constituents among the hits were docked specifically to the inverse agonistbound conformation, 3NY8, using the Glide extra precision (XP) algorithm. Protein-ligand interaction and pose was viewed on the Schrodinger Maestro.

### Validation of Inverse Agonism

Validation of the inverse agonist influence of the lead compounds was done based on the preferential affinity of inverse agonists to the inactive state of the protein. The compounds were docked against the active state, 3P0G [16] and inactive state, 2RH1 [17] of human β2-AR, to determine their preference to either of the receptor states in equilibrium.

### ADMET/Tox Screening

The lead compounds were further screened for their Absorption, Distribution, Metabolism, Excretion and Toxicity using the QikProp program [18].

## Results & Discussion

The various non-covalent interactions between a ligand and the molecular target all aimed at defining the ligand specificity, stability and affinity. Molecular interaction study of phytochemicals from Agemone mexicana and β2-AR carried out in this study is possible by the readily available 3D structure of the protein captured in different conformations on the Protein Data Bank (PDB), particularly to an inverse agonist, ICI-118551 (ICI). Decades before now, study of the molecular mechanisms of inverse agonist was an impossible procedure since basal receptor activity is generally not pronounced, until the construction of constitutively active mutant receptors (CAM) [19] and the overexpression of the wild type receptor [20]. Although we employed an in-silico approach, the high-density expression of β2-AR within the lung cells [21] forms the basis for our plausible submission that this current study is relevant in a live model.

### Molecular Docking

**Figure 2:**
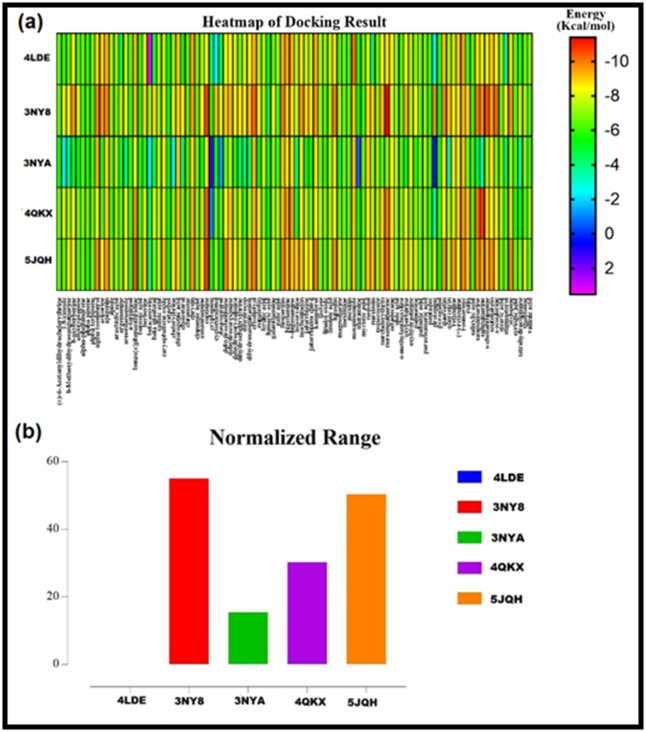
Docking results of the plant constituents against the five protein conformations presented on Heat map (a), and the normalized result, presented on bar chart (b), showing the affinity of the plant constituents to the inverse agonist-bound conformations, 3NY8 and 5JQH.

Virtual screening performed in this study not only reduce the ligand library to compounds with high affinity and/or specificity to the target proteins, but most importantly revealed the suitable protein conformation which is most fitting to the plant constituents. We observed that the plant constituents bind conveniently with lesser ΔG values and higher affinity to both inverse agonist-bound conformations (3NY8 and 5JQH) than the other protein conformations (Figure 2). This is suggestive of the inverse agonist activity of Agemone mexicana's phytochemicals. Although, several groups of compounds were identified in the plant, we, for the sake of this study, focused on the flavonoid members for further molecular interaction profiling and pharmacological scrutiny. Glide extra precision (XP) algorithm is a powerful and more discriminating docking and scoring procedure. Docking of the flavonoid constituents to the inverse agonist-bound protein revealed two flavanones; Eriodictyol (ERI) and Hesperitin (HES) as lead compounds with higher binding affinities of -10.684 kcal/mol glide score and -9.958 kcal/mol respectively, when compared with the standard inverse agonist ICI, which binds with energy of -9.503 kcal/mol.

### Interaction Profiling

**Figure 3:**
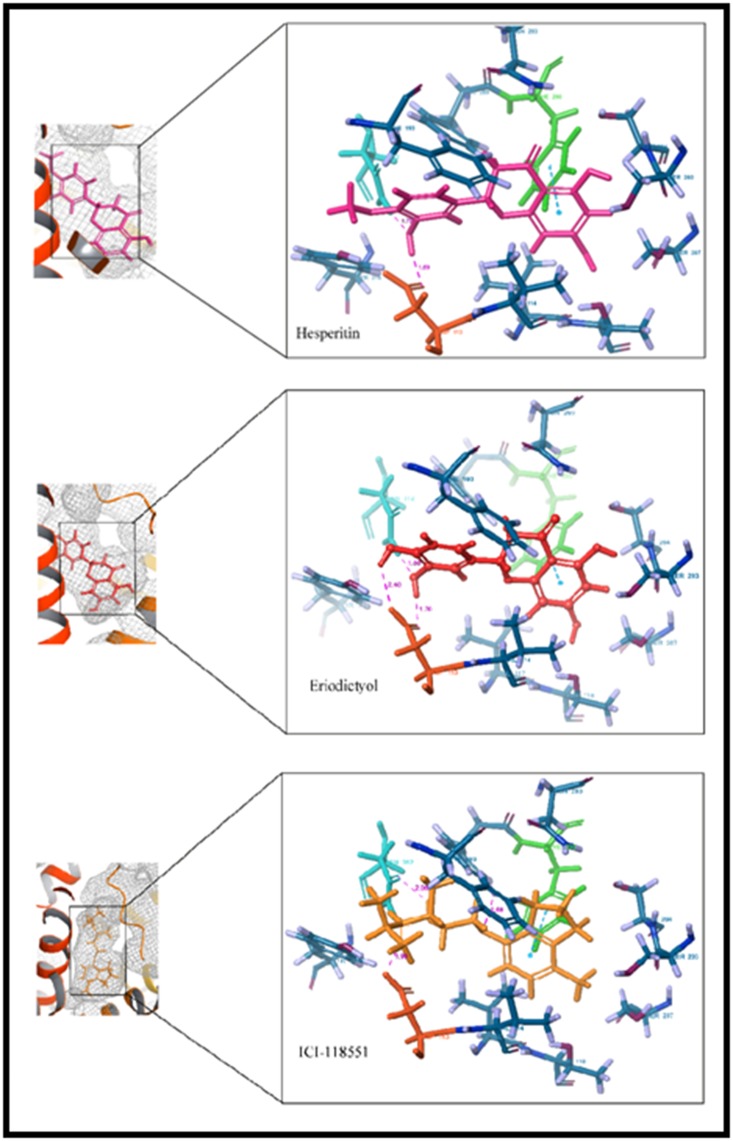
Interaction profile of (a) HES (b) ERI (c) ICI at the β2-AR binding pocket, showing Asp113 (red) and Asn312 (cyan), the key amino acid residues; Phe290 (green) which forms pi-pi stalking, and other interacting residues (blue).

**Figure 4:**
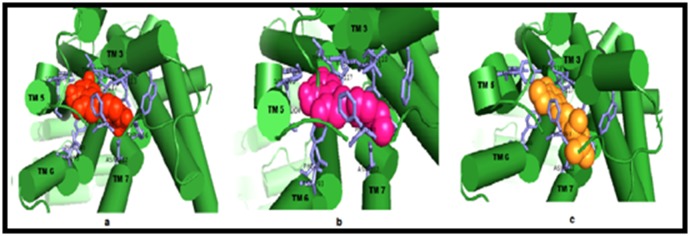
Interaction of ERI (a), HES (b) and ICI (c) with the residues of TM3, TM5, TM6 and TM7 at the β2-adrenergic receptor active site

**Figure 5:**
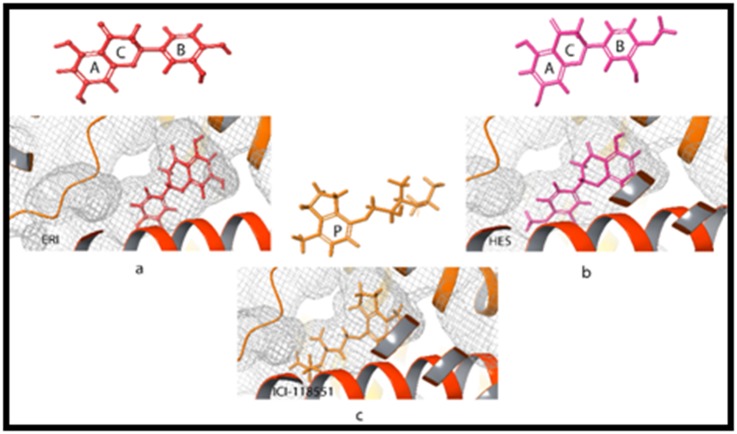
3D structure (above) and binding pose (below) of (a) ERI (b) HES (c) ICI. The binding pose shows the similarity in the orientations of the flavanones and the standard inverse agonist, ICI at the binding pockets of the receptor.

The molecular mechanisms of β2-AR activation or stabilization by the interactions of various ligands have been extensively studied. These studies revealed key amino acid residues necessary for ligand binding. Residues of TM3, TM5, TM6, and TM7, particularly, Asp113 and Asn312, have been reported to be crucial for ligand binding [22]. Interestingly, ERI and HES were found interacting with these key residues at the β2-AR binding pocket (Figure 3 and 4). The orientation of the binding poses of ERI and HES as compared to ICI suggests a similar interaction at the protein's orthostheric site (Figure 5). ICI consists of an ethanolamine backbone connected to a phenyl ring P by an oxymethylene bridge, whereas, the two phenyl rings A and B of the flavanones were joined together by another heterocyclic ring C. The phenyl ring A of ERI and HES, as well as the phenyl ring P of ICI, were buried deep in the binding pocket and enveloped within hydrophobic side chains. These phenyl rings were found to contribute to the stability of the compounds at the hydrophobic pocket by forming pi-pi stacking with Phe290.

**Figure 6:**
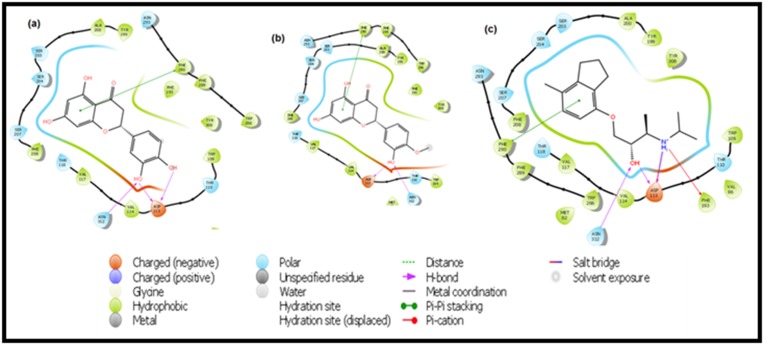
2D interaction view of ERI (a), HES (b) and ICI (c), showing hydrogen bonding, pi-pi stalking and interacting amino acid residues at the binding pocket.

Hydrophobic interactions are essential for activating or stabilizing the receptor. ERI and HES shared hydrophobic interactions with residues of TM5 (Tyr199, Ser203, Ser207) and TM6 (Trp286 and Phe290), interactions that are consistent with initial report [22]. These interactions define the stabilizing efficacy of inverse agonists to the constitutive active receptor. ERI and HES also formed hydrogen bonds with the same residues as ICI, i.e. Asp113 and Asn312. The amino group and hydroxyl group of the ethanolamine backbone of ICI both donate protons to Asp113, while the hydroxyl group accepts proton from Asn312. Same hydrogen bonding is also observed in ERI, whose C19 and C21 hydroxyl groups both donate protons to Asn113, and the C19 hydroxyl group accepts a proton from Asp312. HES also shows the same hydrogen-bonding pattern, with the exception of C21 proton donation to Asp113 (Figure 6).

### Validation of Compound Inverse Agonism

**Figure 7:**
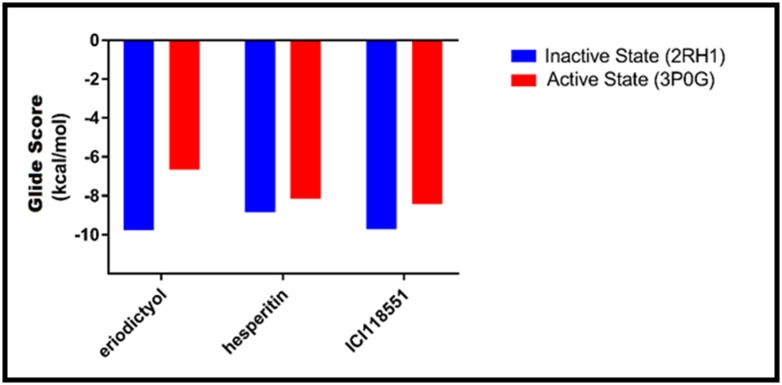
Graph of binding affinity of ERI, HES and ICI to the active and inactive state of the β2-AR, showing ERI with the highest preference for the inactive state of the receptor.

The ability of inverse agonists to bind with preference to the inactive state of GPCRs is required for receptor stabilization (Figure 1). ERI and HES when docked to the active (3P0G) and the inactive (2RH1) states were confirmed to be inverse agonists of the human β2-AR (Figure 7). Result established the selective binding mode of these compounds to the inactive receptor. ERI was found to bind to the inactive protein with the highest affinity of -9.777 kcal/mol glide score, and lowest affinity of -6.650 kcal/mol, to the active state compared to ICI and HES. The order of preference as follows; ERI > ICI > HES, shows ERI, as a more potent inverse agonist of the β2-AR than ICI and HES.

### ADME/Tox Screening

The pharmacokinetics properties of drug, which include Absorption, Distribution, Metabolism, and Excretion, are important parameters, which describe drug disposition and efficacy. These criteria influence the amount of drug reaching the desired target receptor, and hence, the drug pharmacological activity. The Lipinski Rule of Five (ROF) summarizes the molecular properties responsible for a compound to be orally active and drug-like [23]. The rule permits a molecular weight < 500Da, hydrogen bond donors ≤5, hydrogen bond acceptors ≤10 and octanol-water partition coefficient (logP) <5. Compounds, which therefore obey this rule, tend to have lower attrition rates during clinical trials, and as such, are good drug candidate with high possibility of reaching the market. Early detection of compound toxicity before synthesis and marketing is likewise imperative to save cost and time in the drug discovery pipeline, according to the maxim 'fail early, fail fast, fail cheaply'. ERI and HES proved to be drug-like and orally active, obeying the ROF, with zero number of violation (Table 2). Also, the predicted qualitative human oral absorption, which scale 1 (low), 2 (medium) and 3 (high), showed that HES, like ICI, has a high absorption rate, while ERI has a medium absorption rate. QPlogKHsa, (Normal range -1.5 to 1.5) (Ntie-Kang 2013), predicts the binding of drugs to human serum proteins (albumin). Based on the assumption that only free drugs can cross membranes to bind the intended receptor, low QPlogKHsa value is therefore required. ERI and HES bind less with serum proteins, with QPlogKHsa values of -0.195 and 0.004 respectively, which shows that these compounds are more likely to reach and bind the β2- AR receptor as to elicit the desired response. The compounds were also observed to have low activity on the central nervous system (CNS), with low prediction of blood-brain barrier penetration (QPlogBB) of -1.901 and -1.517, which fall within the normal range -3.0 to 1.2 [24]. Since these compounds are peripherally acting compounds, it is therefore required that they have no/low activity on the CNS, to avoid a major neurological insult.

## Conclusions

So far, this current study has shown the potency of two flavanones from Agemone mexicana as suitable inverse agonists of the β2-AR in the treatment of asthma. Molecular docking simulation, interaction profiling and ADME/Tox screening proved ERI and HES as suitable candidates that could be beneficial in the long-term palliative care of asthma. This study focuses on the flavonoid constituents of the plant, we therefore suggest that further pharmacological enquiry be made on the potency of the other plant constituents. Also, in-vitro and in-vivo studies need to be conducted to corroborate the anti-asthmatic potentials of ERI and HES.

## Figures and Tables

**Table 1:** Showing the respective crystallized ligand of 4LDE, 3NY8, 3NYA, 4QKX and 5JQH and the grid coordinates generated around them.

Proteins	Crystallized Ligand	x	y	z
4LDE	BI167107	-13.5	-21.56	-54.13
3NY8	ICI-118551	-2.68	0.95	8.64
3NYA	Alprenolol	2.13	-1.32	7.08
4QKX	35V	5.09	-21.92	-51.32
5JQH	Carazolol	6.65	-33.95	-60.93

**Table 2:** Showing docking result and pharmacological properties of Eriodictyol, Hesperitin and ICI-118551

S/N	Name	Glide Score kcal/mol	Dock Score kcal/mol	M.W. g/mol	ROF Viol	HOA	QlogBB	QlogKHsa
1	Eriodictyol	-10.684	-10.679	288.256	0	Medium	-1.901	-0.195
2	Hesperitin	-9.958	-9.952	302.283	0	High	-1.571	0.004
3	ICI-118551	-9.503	-9.501	277.406	0	High	0.059	0.299

## Abbreviations

β2-AR: β2- adrenergic receptor
ERI: Eriodictyol
HES: Hesperitin
ICI: ICI-118551
ADME/Tox: Absorption, Distribution, Metabolism, Excretion and Toxicity
GPCR: G-protein coupled receptor
CNS: Central Nervous System
